# Editorial: Decoding cancer complexity with artificial intelligence and molecular level studies: informatics-driven approaches for therapeutic innovation

**DOI:** 10.3389/fonc.2026.2008527

**Published:** 2026-10-28

**Authors:** Chandrabose Selvaraj, Krishnendu Bera, Senthilkumar Krishnamoorthi, Sajitha Lulu S

**Affiliations:** 1 CSRDD Lab, Bioinformatics Division, Department of Marine Biotechnology, AMET University (Deemed to Be University), Chennai, Tamil Nadu, India; 2 Living Systems Institute, Stocker Road, University of Exeter, Exeter, United Kingdom; 3 Foundation Traditional Chinese Medicine Research Institute for Age-related Diseases (MFRI), School of Chinese Medicine, Hong Kong Baptist University, Hong Kong, Hong Kong SAR, China; 4 Integrative Multiomics Lab, School of Bio Sciences and Technology, Vellore Institute of Technology, Vellore, Tamil Nadu, India

**Keywords:** artificial intelligence, breast cancer, cancer, mRNA vaccine, multi-omics

Cancer persists as a leading cause of mortality worldwide, underscoring the urgent need to decipher its molecular complexity for improved detection, prognosis, and treatment. Cancer is characterized by numerous and sequential events, including epigenetic regulation, alteration in genes, and proteins that highly interact with the surrounding environment (1). The integration of artificial intelligence (AI), machine learning (ML), and advanced bioinformatics is currently revolutionizing our understanding of cancer biology, uncovering novel biomarkers, elucidating therapeutic response mechanisms, and accelerating the development of personalized therapies (2). The special issue*, “Decoding Cancer Complexity with Artificial Intelligence and Molecular Level Studies: Informatics-Driven Approaches for Therapeutic Innovation”* compiled the contributions of 13 different studies dealing with emerging topics and various computational approaches that are transforming modern cancer research. These contributions significantly cover the important areas, including disease diagnosis, biomarker identification, toxicity assessment, rational design and engineering of cancer vaccines, disease prognosis, and prediction of therapeutic responses. The core concept emerging from the current issues is the shift from single-modality analysis to multimodal and biologically contextualized intelligence.

Huang et al. emphasize the AI-driven digital pathology in hepatobiliary and pancreatic tumors; they have discussed the significance of whole-slide imaging and the role of deep learning in disease diagnosis, prognosis, molecular prediction, and therapeutic response assessment. Majorly they have discussed the crucial role of data heterogeneity, interpretability, prospective validation, and incorporation of clinical workflows and also highlighted the insufficiency of superior algorithmic performance in clinical translation. This concept is further supported by Ding et al. reporting that TriBind-Mamba is a tri-model framework that incorporates the clinical knowledge, whole-slide image, and transcriptomic information via selective state-space models. In computational oncology, the incorporation of pathway-level molecular representations and clinical knowledge prompting signifies an extensive advancement; hence, there is a need to generate a new model to contextualize predictive patterns with clinically significant and biologically interpretable frameworks, along with identifying them.

Zhang et al. emphasize the AI-driven multimodal fusion involving radiomics, multi-omics, circulating tumor DNA, clinical variables, and digital pathology, underscoring the potential of cross-modal association in the realms of diagnosis, treatment-response prediction, and prognostic assessment. Peng et al. discussed triple-negative breast cancer and also emphasized the transcriptomics, multi-omics, pathology, and significance of imaging owing to the pronounced biological heterogeneity inherent in these cancer subtypes. Their analysis significantly underscores the importance of maintaining the reliability despite missing modalities, institutional variations for future systems, and shifts in real-world data distribution. Tong et al. have predicted a machine-learning-based mitochondrial gene signature for breast cancer, utilizing vast computational analysis, immune-filtration analysis, tissue validation, drug prediction, single-cell data analysis, molecular docking, and molecular dynamics simulations. Their findings signify the identification of mitochondrial-linked biomarkers, and exploring therapeutic molecules is important for the incorporation of advanced computational prediction with molecular and experimental evidence. Wang et al. progressed the internally validated machine-learning model to predict the early stage of bone metastasis in patients newly diagnosed with prostate cancer. This study identifies the clinical laboratory predictors and performs SHAP-based interpretation to improve model transparency. Along with the development of interactive prediction tools, this report illustrated the potential of informatics to support individualized clinical risk estimations. The authors significantly recognize the need for external and prospective validation as subsequent steps. Allena et al. performed a comprehensive evaluation of quantum machine-learning architectures for breast cancer classification, utilizing clinical and genomic data. Their results are remarkably valuable, as they prove a realistic benchmark rather than asserting a quantum advantage; within the tested simulator and dimensionality constraints, classical models utilizing the complete feature space significantly outperformed the quantum approaches evaluated. This contribution underscores the significance of rigorous benchmarking as novel computational approaches are incorporated into biomedical research. Gopalan and Sundararajan performed an understandable graph-attention framework that incorporates genomics, proteomics, transcriptomics, and chemical descriptors to identify the cancer drug responses. Their analysis significantly correlates the model-derived features importance with pathway enrichment analysis and consistently signifies the association of PI3K/AKT/mTOR signalling pathways. This approach demonstrates that explainability can go beyond ordinary visualization of model decision, which facilitates the generation of mechanistic hypotheses for experimental validation. Guhanraj work on bacteriocins and postbiotic metabolites as effective and potential anticancer agents underscores the incorporation of microbiome biology with AI-based techniques, molecular docking, molecular dynamics, system biology, and nanotechnology. This viewpoint extends the scope of computational oncology beyond conventional small molecule identification, incorporating biologically diverse microbial products and their interaction with tumors.

Sharma et al. developed a ModernBERT-based molecular language model, ChemModernBERT, to predict the LD50 values with the assessment of over 1.8 million SMILES strings using standardized datasets. The study demonstrated the potential of molecular language models in computational toxicity assessments and chemical prioritization. Furthermore, the research underlines the importance of distinguishing between acute toxicity prediction and the broader biological processes implied in carcinogenesis. Srivastava summaries AI-enabled approaches for engineering mRNA cancer vaccines, which integrate neoantigen prediction, MHC binding, antigen processing, T-cell recognition, codon optimization, UTR engineering, mRNA structural modeling, and lipid nanoparticle design. This work emphasizes the capability of computational intelligence to connect tumor-specific molecular data with coherent vaccine design and personalized immunotherapy.

Li et al. conduct an inclusive mapping of the global academic landscape concerning AI applications in basal cell carcinoma, exposing a significant surge in research activity since 2019. They discover deep learning-based dermoscopic and digital pathology treatments as predominant themes. Their bibliometric analysis extends valuable insights into the rapid development of computational oncology across various disease areas. Zhang et al. explore pathomics-derived tumor-infiltrating lymphocyte scores in colorectal cancer whole-slide images from multiple cohorts. This study tackles the merging of computational pathology, tumor immunity, and population diversity. The multicohort design is specifically appropriate to the key target of developing AI systems efficient of acquiring biological variation, rather than simply learning patterns regulated to a single institutional population.

Overall, the papers from this issue highlights the computational oncology, doesn’t depend only on the complex algorithms, but also requires the integration of diverse biological data with clinically relevant samples. Digital pathology reveals spatial features, genomics and transcriptomics explain molecular changes, clinical data provides patient-specific context, and computational chemistry helps understand therapeutic and toxicological properties. AI can integrate these different dimensions into a unified framework. Among these studies, few limitations like data heterogeneity, limited external validation, interpretability, reproducibility, and the lack of prospective clinical evaluation remains notable. Future research should therefore focus on multicentre validation, standardized data generation, biologically informed interpretation, privacy-preserving collaboration, and clinical evaluation. The future of oncology lies not simply in applying AI to cancer, but in integrating AI with cancer biology to develop systems that are explainable, clinically relevant, experimentally testable, and robust across diverse cancer populations.
